# A Benchmark Dataset of Chinese Development Finance with Climate Relevance and SDG Annotations from 2000–2021

**DOI:** 10.1038/s41597-026-06605-9

**Published:** 2026-01-20

**Authors:** Ji Qi, Yayun Tang, Zhenyi Zhang, Yu Wang, Haoqi Qian

**Affiliations:** 1 Institute for Global Public Policy, Fudan University, Shanghai, 200433 China; 2 Fudan Institute for Advanced Study in Social Sciences, Fudan University, Shanghai, 200433 China; 3 LSE-Fudan Research Centre for Global Public Policy, Fudan University, Shanghai, 200433 China; 4 MOE Laboratory for National Development and Intelligent Governance, Fudan University, Shanghai, 200433 China

**Keywords:** Climate change, Climate change, Developing world

## Abstract

China has become a major provider of climate finance to the Global South, yet systematic and transparent data capturing the full scope of its efforts remains limited. This study presents a refined project-level dataset of Chinese development finance from 2000 to 2021, comprising 20,985 records from the AidData Global Chinese Development Finance Dataset. Using a multi-stage approach, we identify 1,383 climate-related projects, amounting to $421 billion, and assign SDG targets to all entries. Climate relevance and SDG alignment were determined through extensive manual annotation following the Rio Marker and SDG frameworks. To ensure consistency and scalability, we conducted technical validation using pretrained BERT models for climate and SDG classification. The resulting dataset not only enables systematic analysis of China’s evolving international climate finance portfolio and sustainable development commitments, but also serves as a benchmark resource for researchers in climate and sustainable development fields to train or refine advanced language models.

## Background & Summary

Although China, as a developing country, has no international obligation to provide support, it has emerged as a major provider of climate finance to enhance cooperation in addressing climate change, particularly through the South–South Climate Cooperation Fund and the Belt and Road Initiative (BRI). Climate finance generally refers to financial resources mobilized from public, private, and multilateral sources to support climate change mitigation and adaptation efforts, especially in developing countries^1^. It plays a central role in implementing the Paris Agreement and achieving the Sustainable Development Goals (SDGs), making China’s growing engagement particularly significant^2,3^. At COP29 in 2024, the Chinese government announced that it had provided or mobilized over 177 billion yuan (approximately USD 24.5 billion) since 2016 to support climate initiatives in developing countries^4^. During the past decade, Chinese overseas climate finance has surpassed that of most developed countries, placing it among the top global contributors between 2013 and 2018^5,6^.

An increasing number of studies have examined the environmental and strategic impacts of Chinese overseas development finance, especially in the energy sector^7–11^. However, much of this research has concentrated on distinguishing renewable energy from fossil fuel investments, while broader climate finance efforts across diverse sectors have received comparatively limited attention, despite their growing importance in addressing the full scope of climate challenges. One important limitation has been the absence of transparent, project-level data that captures the full scope of Chinese climate finance. Without this data, it is difficult to analyze trends, understand sectoral priorities, or assess how such finance aligns with broader SDGs.

Tracking climate finance and the progress towards SDGs has long been complicated by the lack of a universally accepted definition^12^. The OECD’s Rio Markers provide a reporting system to classify projects based on their relevance to mitigation or adaptation, using a three-tiered marker system. However, a large share of reported projects to the OECD Creditor Reporting System (CRS) lacks detailed descriptions, making external validation difficult. To address this challenge, we apply a content-based filtering approach that follows the Rio Markers classification and incorporates the methodology of the Climate Policy Initiative^13^.

This study builds on earlier efforts to track Chinese overseas development finance, particularly the AidData Global Chinese Development Finance Dataset^14^. Previous estimates of Chinese climate finance have largely relied on keyword searches and broad labels^5,6^. Another stream of research applies the machine-learning model to classify the climate-related projects and label the SDG targets based on their textual descriptions^15,16^. In this study, we introduce a refined dataset that was obtained with cross-verification based on BERT models. This process allows for a more accurate and detailed identification of climate-related projects across sectors.

The dataset presented here provides new opportunities to study how Chinese development finance contributes to global climate and development goals. It enables more precise identification of aid projects linked to mitigation and adaptation and offers a clearer picture of how these projects support specific SDG targets. The data also help reveal how Chinese climate finance has changed over time, including shifts in geographic focus, sectoral priorities, and project timing. Where official donor classifications are missing, this approach offers an alternative based on systematic content analysis and validation. Scholars can explore China’s strategic engagement with recipient countries and its positioning relative to OECD donors^9,17–19^. This data enables further assessment of the environmental and socioeconomic effects of Chinese projects at the local level^8,20^. Besides, by linking projects to specific SDG targets, the dataset can support forward-looking assessments of how Chinese climate finance contributes to sustainable development outcomes in partner countries^21^.

Finally, the methodological framework developed here is transferable to other underreported contexts, enabling cross-country comparability and more comprehensive global assessments of climate finance^22^. As a benchmark dataset, our manually labelled records offer a transparent, reproducible ground truth that supports training, fine-tuning, and validating machine-learning models for climate- and SDG-related classification, while providing a stable reference point for method comparison and error analysis. Benchmark resources of this kind have been shown to accelerate methodological progress, improve out-of-sample robustness, and enable consistent evaluation across tasks and domains in adjacent fields^23,24^. Together, these contributions aim to advance methodological development, strengthen empirical research, and support evidence-based policy design in global climate finance and sustainable development agendas.

## Methods

In this section, we provide an overview of the database’s construction. The Chinese development finance dataset with climate marker and SDG categorizations is sourced from AidData’s Global Chinese Development Finance Dataset (Version 3.0)^14^, covering 20,985 projects across 165 low- and middle-income countries from 2000 to 2021. The Chinese climate-related development finance dataset covers 1,383 projects in 145 countries and regions from 2000 to 2021.

**Fig. 1:**
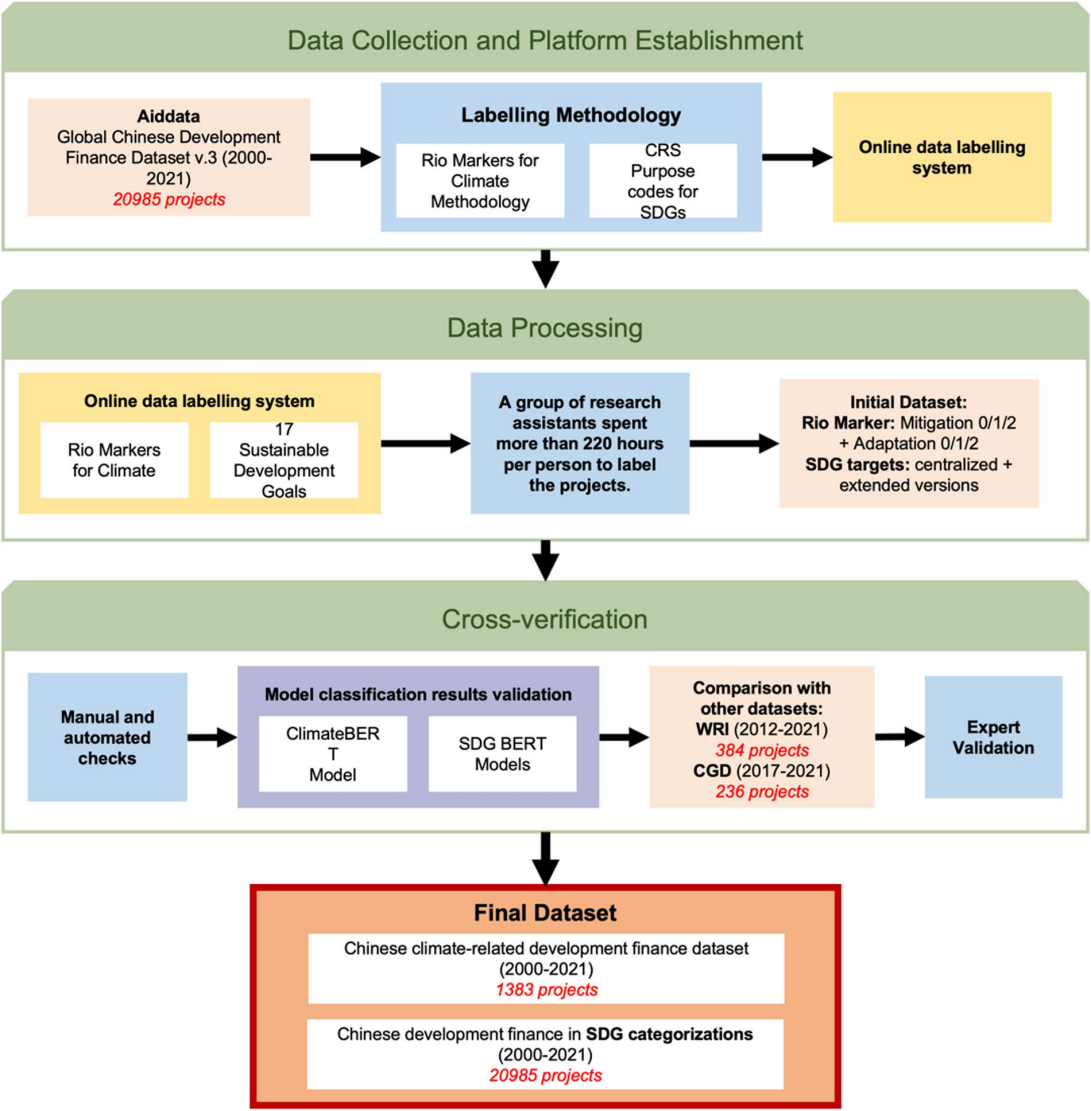
Data processing steps for the Chinese development finance with Rio Marker and SDG Markers. Orange: datasets; blue: manual labelling and methodology; yellow: online labelling system; purple: model validation.

The process of constructing the database involved three main steps (Fig. 1): (1) Constructing the online data labelling platform for project-level Chinese development finance data and building up labelling methodology; (2) Labelling 20,985 projects in Rio Markers and SDGs; (3) Validating the integrated results through manual checks, comparing with the predictions by the pretrained BERT models and existing datasets, and merging the final data with project information to generate the database.

### Data collection and platform establishment

We first obtained the project-level data of the Global Chinese Development Finance Dataset (Version 3.0) from the Aiddata website. The project information includes the project name, project description, commitment year, recipient, sector, flow type, and financing details. All the project contents are in English and are well organized in standard forms.

Next, we developed an online labelling platform that presented key project information on the interface, with the primary purpose of enabling experts to independently assign classifications. To assess climate-relevance, we mainly drew on the guidelines set by the OECD Development Assistance Committee’s Rio Markers for Climate Methodology and complemented with the Climate Policy Initiative’s methodology where relevant^13,25^. We also compared our approach to that taken previously by the World Resource Institute (WRI) and the Center for Global Development (CGD), ultimately making several significant expansions in the sectors and activities covered within our own assessment^5,6^. Regarding the SDG markers, we followed the SDG target information outlined in the 2030 Agenda for Sustainable Development, as well as Aiddata’s CRS Purpose codes and SDGs^26,27^. A labelling sample for the project can be found in Table 1 (See also Supplementary Tables 1 and 2 for the labelling methodologies).

**Table 1:** An example of the project labelling sample in Rio Marker and SDG Marker.

Project Description	Sector	Rio Marker (Overall, Mitigation, Adaptation)	SDG Marker
Training of water-saving and drought-resisting rice technology	Agriculture, forestry and fishing	2,0,2	2,6,13
Loans that incentivize better climate change risk analysis and increase its funding for green projects	Banking and financial services	2,2,2	7,13
Private investment in electric/hybrid vehicles production	Business and other services	2,2,0	9,11,13
Remote sensing satellite serving for meteorology, agriculture and natural disaster prevention purposes	Communications	1,0,1	9,13
Food aid for disaster relief for flood victims	Developmental food aid/food security assistance	2,0,2	1,2,13
River deepening and sluice gate upgrading to reduce flooding	Disaster Prevention and preparedness	2,0,2	6,11,13
Rural solar power photovoltaic equipment donation to university	Education	2,2,0	4,7,13
Recovery plan for the tsunami-affected area and promote eco-tourism	Emergency response	1,0,1	8,11,13,14/15
Solar photovoltaic power generation systems to rural areas	Energy	2,2,0	1,7,9,13
Scientific investigation of climate change and water resources	General Environmental Protection	2,2,2	6,13
Electric vehicle donation for public duties	Government and civil society	1,1,0	11,13
Upgrade hospitals and local health centers in areas affected by flooding	Health	1,0,1	3,11,13
Construction of a photovoltaic power station to supply electricity to factories, schools and homes	Industry, mining, construction	2,2,0	7,9,11,13
Social housing unit and infrastructure that includes roads, water supply and drainage system, electric light systems, etc.	Other social infrastructure and services	1,1,1	6,7,9,11,13
Build bridges to provide access to the flooded villages	Reconstruction relief and rehabilitation	1,0,1	9,11,13
New energy buses	Transport and Storage	2,2,0	11,13
Water resource research project related to climate change mitigation and hydropower development	Water supply and sanitation	2,2,1	6,7,9,11,13

### Data processing

After all projects were uploaded to the online annotation platform, a team of postgraduate students specializing in public policy, with training in climate policy, undertook the labelling process. The annotation process required over 750 hours to complete the labeling of 20,985 projects. Prior to the formal labelling phase, each student completed a practice set ranging from 100 to 500 projects, during which labelling standards were clarified and aligned in accordance with a detailed methodology guide. Projects were randomly assigned by the platform, and each was independently reviewed by at least three annotators. The inter-annotator agreement statistics are included in the final dataset. A high level of consistency was achieved, with 85.38% of projects showing complete agreement among the three annotations.

Following the initial round of labelling, the research team examined projects with inconsistent classifications. Research assistants proposed revisions based on internal discussion, and unresolved cases were escalated to senior researchers with expertise in climate policy for final judgment. This multi-stage process aimed to ensure consistency and transparency in project classification.

**Fig. 2:**
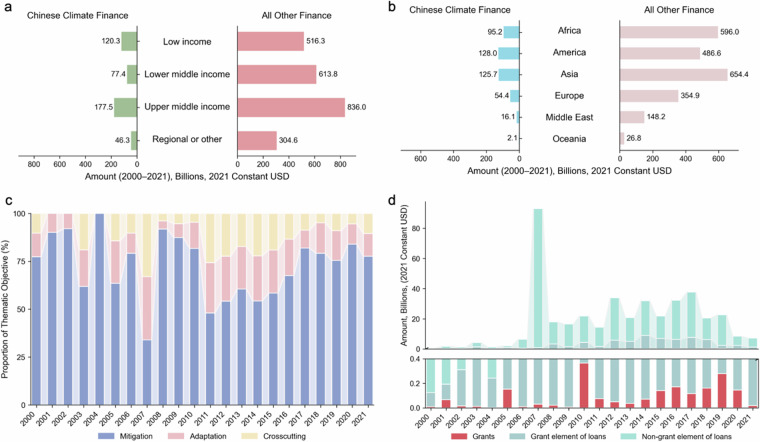
Chinese international climate finance to developing countries from 2000 to 2021. All other finance refers to Chinese development finance, excluding climate finance. Data are based on the cross-verified results between manual annotations and BERT-model validation. (**a**) By recipients’ income groups; (**b**) By recipients’ regions; (**c**) By thematic objectives of the project; (**d**) By instruments and grant elements.

Each project was evaluated for its relevance to climate finance based on two primary themes: mitigation and adaptation. Mitigation refers to efforts that reduce greenhouse gas emissions or enhance carbon sinks, while adaptation includes actions that address the impacts of climate change by reducing vulnerability or improving resilience. In line with the Rio Marker system, projects were scored on a three-point scale: 0 (“not targeted”), 1 (“significant objective”), and 2 (“principal objective”). A score of 2 indicates that climate change mitigation or adaptation is the main purpose of the project. For example, a project aimed at deploying renewable energy technologies would typically receive a score of 2 for mitigation, as reducing emissions is its central objective. A score of 1 reflects projects where climate action is an important but secondary aim. For instance, a water infrastructure initiative that enhances climate resilience or a disaster risk reduction program with climate adaptation components would typically be classified under this category. In addition to explicit climate-focused activities, the annotation also identified projects with broader environmental objectives that may yield climate co-benefits. These include initiatives related to biodiversity conservation, sustainable land use, and waste management. Such projects were typically classified as having a “significant” climate objective. Figure 2 provides an overview of the 1,383 projects identified as climate-related between 2000 and 2021, based on the final dataset obtained after cross-verification between manual annotations and BERT-model predictions.

To align project classification with the SDGs, we assigned each project to relevant SDG targets based on its description and its stated contribution to the goal. Projects were linked to the most pertinent SDGs that could be considered as having a “significant” association with the project’s objectives. Since each project received three independent labels from different research assistants, we present two versions of SDG classification. The first, referred to as the centralized version, includes SDGs that appeared in the intersection of at least two out of the three individual labels. The second, or extended version, reflects the full set of SDGs identified in at least one of the three annotations.

**Fig. 3:**
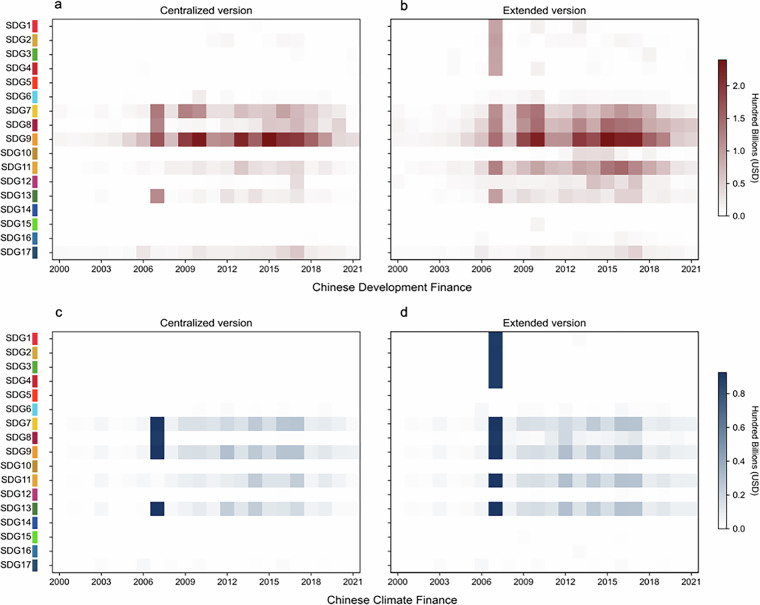
Cumulative allocations of Chinese Development Finance and Climate Finance (2000–2021) across the 17 SDGs. Climate finance estimates are based on cross-verified results from manual and model-assisted annotation, while SDG annotations rely exclusively on manual labeling. The centralized version includes SDGs that appeared in the intersection of at least two out of the three individual labels. The extended version reflects the full set of SDGs identified in at least one of the three annotations.

A key challenge in this process is the overlapping nature of many SDG targets. Advancing one target often contributes to progress in others, but our classification prioritizes those goals most directly related to the project’s activities. Projects may be linked to multiple SDGs for two main reasons. In some cases, a single activity is relevant to more than one target—for example, both SDG 3.7 and SDG 5.6 relate to access to sexual and reproductive healthcare. In other cases, a project includes distinct components that align with different goals, such as a program that delivers both water and sanitation services (SDG 6) and vocational training (SDG 8). Figure 3 illustrates the distribution of Chinese development finance and climate finance across the 17 SDGs.

### Cross-verification

We conducted a multi-step cross-verification of the annotated results, as detailed in the Technical Validation section. Following an initial round of manual review and automated checks, we applied two pretrained language models—ClimateBERT and SDG-BERT—to further classify the projects. The ClimateBERT model was used to determine whether a project could be considered climate-related, while the SDG-BERT model generated probability scores for each SDG based on project descriptions. To finalize the results, we applied defined probability thresholds to interpret the outputs of the SDG-BERT model.

We then compared the predictions from both BERT models, as well as classifications from existing datasets, with the labelled data generated in this study. Under the assistance of experts, we manually reviewed and adjusted 3.18% of the cases, corresponding to 667 projects, after cross-checking the annotation results. Finally, we aggregated the Rio Marker scores and SDG classifications for each project and integrated them with additional project-level metadata. This process allowed us to compile a structured database of Chinese climate-related development finance, including both Rio Marker and SDG-based categorizations.

## Data Records

The datasets are available through Figshare^28^. We provide two datasets of Chinese development finance covering the period from 2000 to 2021. These datasets are available in Excel format to ensure compatibility with common analytical tools:

- Chinese climate-related development finance dataset (2000–2021): This dataset includes details such as the year, project ID, recipient, project title, project description, project finance amounts, climate-related mitigation/adaptation marker, funding agency, and types of finance (loans, grants, etc.).
- Chinese development finance dataset in SDG categorizations (2000–2021): The dataset includes details such as the year, project ID, recipient, project title, project description, project finance amounts, SDG marker (two versions: centralized and extended), funding agency, and types of finance (loans, grants, etc.).

## Technical Validation

### Model validation and Comparisons

To validate the annotated results, we employed ClimateBERT (https://huggingface.co/climatebert/distilroberta-base-climate-f) and a suite of SDG-BERT models, including the Aurora SDG mBERT model, the Aurora SDG multi-label mBERT model, and the Elsevier SDG multi-label model (https://aurora-universities.eu/sdg-research/classify/, https://zenodo.org/records/7095784)^29–31^. ClimateBERT is a pretrained language model designed for text classification tasks related to climate change. It was originally trained on more than two million climate-related texts from academic and policy sources and achieved an F1 score of about 0.99 in benchmark evaluations.

To adapt ClimateBERT to the context of development finance, we fine-tuned it on a manually labeled subset of 1,500 project records from the Chinese Development Finance dataset (2000–2021). The task was to determine whether each project was climate-related, following the Rio Marker framework. To ensure balanced representation, 750 climate-related and 750 non-climate-related projects were randomly selected from the full AidData-based dataset, then shuffled and divided into training and validation sets with a 90:10 split. Fine-tuning was performed using the AdamW optimizer with a learning rate of 2 × 10−⁵, a weight decay of 0.01, and a linear learning-rate warmup schedule. As shown in Table 2, the model was trained for five epochs, and the checkpoint with the highest validation F1 score was retained as the final version. The best performance was achieved in the fourth epoch, with an F1 score of 0.918, indicating a balanced tradeoff between detection accuracy and coverage.

**Table 2:** Performance metrics of the fine-tuned ClimateBERT model across five epochs.

Epoch	Training Loss	Validation Accuracy	Precision	Recall	F1 score	Best model saved
**1**	0.5006	0.8933	0.9155	0.8667	0.8904	√
**2**	0.2348	0.9133	0.9306	0.8933	0.9116	√
**3**	0.1477	0.9133	0.9079	0.9208	0.9139	√
**4**	0.0931	0.9133	0.8690	0.9733	0.9182	√
**5**	0.0795	0.9067	0.9296	0.8800	0.9041	—

Using this customized version of ClimateBERT, we identified 2,640 projects as climate-related between 2000 and 2021, each with a classification probability above 90 percent. While the model performs well in binary classification, indicating whether a project is climate-relevant, it does not provide the tiered classification system used in the Rio Marker framework, which distinguishes among “principal,” “significant,” and “not targeted” objectives.

To further assess the quality and consistency of our data, we compared our dataset with two existing datasets that also aim to identify Chinese climate-related development finance. Both of these prior datasets rely on keyword-based classification applied to the AidData Global Chinese Development Finance database. The first, published by CGD, identifies 236 climate-related projects between 2017 and 2021^6^. The second, published by WRI, includes 384 projects from 2012 to 2021, though it limits classification to five core sectors and selected multi-sectoral activities^5^. A direct comparison with the WRI dataset further showed that our annotations covered nearly all projects identified by WRI, confirming the consistency and completeness of the manually labeled data. In addition, our approach identified several relevant projects that were not captured by WRI, likely due to its keyword-based screening. This suggests that our content-based method provides a more comprehensive recognition of climate-related projects.

**Fig. 4:**
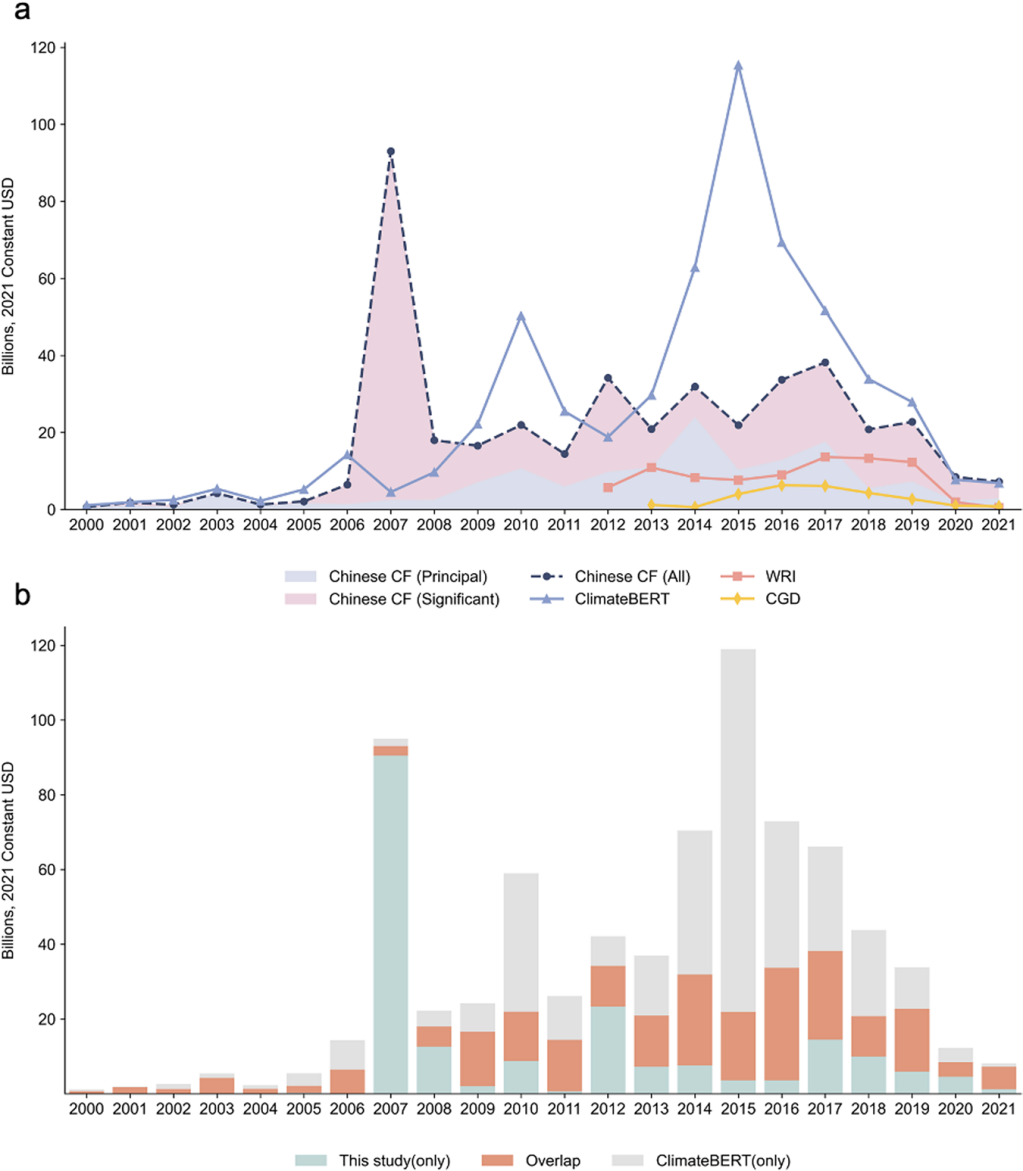
Comparative performance with existing datasets and the ClimateBERT model.

Unlike these keyword-based approaches and the binary outputs of pretrained BERT models, our dataset offers more detailed classifications following the Rio Marker methodology. It assigns projects to climate change mitigation or adaptation categories using a three-tiered scoring system, capturing both “significant” and “principal” objectives. These results highlight the added value of manual annotation and expert review, which not only enhance the interpretability of the data but also allow for a more granular understanding of climate relevance across diverse sectors. Figure 4 compares our climate finance dataset with the predictions from the ClimateBERT model and the existing datasets. Table 3 presents examples of projects with differing labeling outcomes between the ClimateBERT model and our study.

**Table 3:** Examples of differing labelling outcomes between ClimateBERT and this study.

ID	Description (Condensed)	Commitment Year	Financial Amount (2021 Constant USD)	ClimateBERT labeled	Rio marker in this study
17957	China has committed $50 billion to the China–Venezuela Joint Fund, co-financed by China Development Bank and Venezuela’s FONDEN, administered by Bandes. The facility—comprising Tranches A, B, C, and a Long Term Facility—can be repaid in cash or oil via long-term supply contracts with ChinaOil. Since 2007 it has financed major infrastructure, industry, and energy projects, including the Valencia, Maracaibo, and Los Teques/Caracas metro systems; the III Bridge over the Orinoco; the Tinaco–Anaco railway; irrigation and dam rehabilitation; cement and seamless pipe plants; and multiple power plants such as TermoZulia II, IV, V, La Cabrera, TermoCarabobo II, India Urquia, and Juan Bautista Arismendi. Other works include Tocoma Hydroelectric Power Plant, five additional thermal plants, agricultural development schemes, industrial upgrades, and social programs. The table below lists identified projects under this facility alongside their Rio marker and SDG labels.	2007	89963219144	No	1,1,1
15080	In October 2010, China Development Bank, Bank of China, China Eximbank, and ICBC signed MoUs with Reliance Power Limited (R-Power) worth $12 billion. These credit lines were meant to fund the purchase of Chinese equipment (Boilers, Generators, and Turbine Procurement) from Shanghai Electric and support the construction of energy projects in India. One of the projects supported under this agreement is the Sasan Ultra Mega Power Project (SUMPP) located in Madhya Pradesh (see Project #54362).	2010	16712723230	Yes	0,0,0
13014	Between July 2011 and mid-2012, a severe drought affected the entire East African region causing severe food shortages in countries like Djibouti. On 4 January 2012, the Chinese government delivered a food aid package worth $10 million USD to Goubetto village in Djibouti. The donation included flour and oil.	2012	11741235.58	No	1,0,1
9906/9929	On April 24, 2015, during the 60th anniversary of the Asian–African Conference, Chinese officials signed a memorandum of understanding for a US$50 billion syndicated loan for infrastructure development in Indonesia, with US$20 billion from ICBC and US$30 billion from China Development Bank. The funds target Indonesian state-owned enterprises such as Perusahaan Listrik Negara (PLN), Aneka Tambang, Adhi Karya, and Pelabuhan Indonesia II. PLN is to receive US$10 billion for transmission lines and multiple power plants, while other SOEs will use the financing for projects such as a smelting plant, the trans-Sumatra toll road (Bakauheni–Terbanggi Besar section), high-speed rail, Light Rail Transit, and Sorong Harbor. A portion is also designated for foreign trade.	2015	22445511391.58 + 33668267087.37	Yes	0,0,0
10157	On December 9, 2015, China Development Bank agreed to a US$15 billion oil prepayment facility with the Government of Angola, to be repaid through oil sales. The facility was used to recapitalize Sonangol, prepay existing debts, and finance a range of public investment projects. Chinese firms undertook most of the work, with at least 20% subcontracted to local companies. Key projects included the construction of a 400 km Laúca–Huambo power transmission line, a dam in Kwanza Norte for hydropower generation, and domestic electricity connections to communities, along with multiple provincial water supply systems and irrigation schemes. The facility also supported major transport infrastructure, including rehabilitation and construction of national roads such as EN100 and EN110/EN280, the Cuito Road, and the Sambizanga–Luanda route, as well as works at Cabinda airport. Social and educational infrastructure formed another major component, with rehabilitation of hospitals and medical centers, expansion of Cabinda University, construction of schools, universities, and training colleges, and social housing developments. Industrial projects included new industrial hubs and integrated urban infrastructure in Sumbe, along with port and terminal works. Known as the “China Credit Line,” the facility was designed to finance 155 public investment projects across nine sectors, of which 34 were energy and water projects, 33 were construction projects, and 55 were education projects, with Luanda and Huambo provinces receiving the largest shares of funding.	2015	1122275570	Yes	0,0,0

The SDG-BERT models allow us to link project descriptions to relevant SDGs by leveraging different model architectures and training datasets. The Aurora SDG mBERT and Aurora SDG multi-label mBERT models were trained on 1.4 million scientific abstracts, using SDG definitions based on the Aurora SDG Queries v5. Both models generate probability scores for all 17 SDGs. While their outputs are broadly consistent, minor differences arise due to variations in model structure and the balance of training data. Additionally, we used the Elsevier SDG multi-label mBERT model, which was trained on a large corpus of abstracts using the Elsevier SDG Queries^31^. Like the Aurora models, it produces a probability score between 0 and 1 for each goal, reflecting the likelihood that the project relates to a given SDG.

**Fig. 5:**
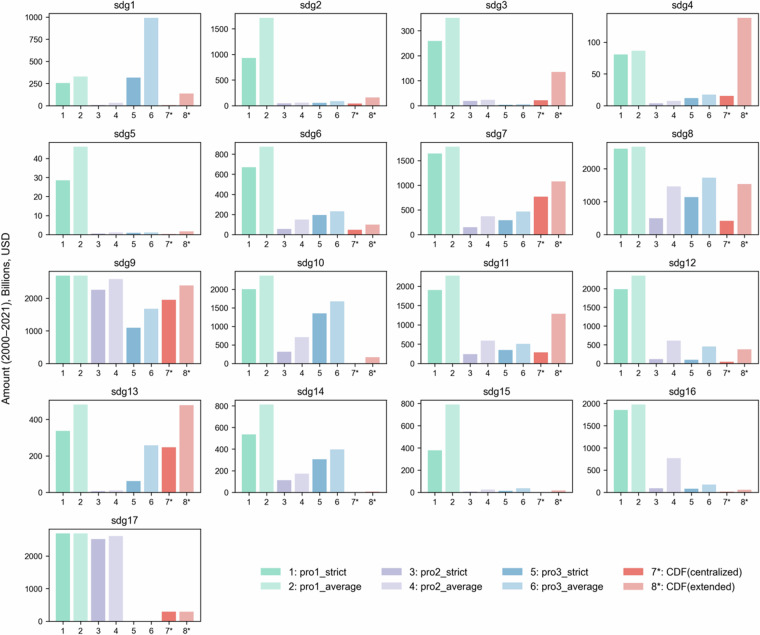
Comparative performance with SDG prediction models. Three SDG classification models are evaluated: pro1 (Aurora SDG mBERT), pro2 (Aurora SDG multi-label mBERT), and pro3 (Elsevier SDG multi-label model). For pro1 and pro2, two thresholding strategies are applied: “average” denotes predictions exceeding 5.88% (i.e., 1 out of 17 SDGs), and “strict” corresponds to 11.76% (i.e., 2 out of 17). As pro3 excludes SDG17, thresholds are adjusted to 6.25% (1/16) and 12.5% (2/16), respectively. Two variants of the Chinese Development Finance (CDF) with SDG Markers are shown: a centralized version and an extended version developed in this study.

While their general patterns are consistent, Fig. 5 highlights several systematic differences. Overall, the pro1 model tends to overestimate SDG relevance, producing higher probability scores across most goals compared to pro2, pro3, and our manually annotated results. In contrast, pro2 and pro3 yield more conservative outputs, aligning more closely with the human-labelled dataset in aggregate, though individual discrepancies remain for certain SDGs such as SDG 4 (Quality Education) and SDG 13 (Climate Action). These divergences underscore that the models’ predictions are sensitive to their architectures and training distributions, particularly in cases where project descriptions include overlapping or ambiguous development objectives.

Because of these inconsistencies, a secondary cross-verification step was essential. We compared the outputs from all three model configurations with our manual annotations to identify stable, interpretable classifications. Table 4 presents representative examples of where the models disagreed with one another and with expert-labelled results. This comparison illustrates that, despite the models’ strong general performance, manual annotation remains the most reliable benchmark, especially for complex multi-sectoral projects where contextual understanding is critical.

**Table 4:** Examples of the differing labelled outcomes between SDG BERT models and this study.

SDG	Main Content of the Project (based on the Description)	SDG BERT pro1-strict	SDG BERT pro2-strict	SDG BERT pro3-strict	SDG Marker in this study (Centralized)
1:No Poverty	A COVID-19-era bilateral debt forgiveness agreement under the G-20 Debt Service Suspension Initiative to increase its support of African countries during hardship (ID: 88157)	Relevant	Not relevant	Relevant	Not relevant
4: Quality Education	A grant-funded educational infrastructure project for a modern public service college headquarters in Guyana (ID: 5382)	Relevant	Not relevant	Not relevant	Relevant
5: Gender Equality	A 298.6 million euro public-private partnership project, partly funded by Industrial and Commercial Bank of China, to build and operate the 2,060-bed Izmir Bayrakli Integrated Health Campus in Turkey (ID: 9320)	Relevant	Not relevant	Relevant	Not relevant
10: Reduced Inequalities	A large-scale, Chinese-financed oil refinery and petrochemical complex development project in Nigeria (ID: 15304)	Relevant	Not relevant	Relevant	Not relevant
13: Climate Action	A massive, Chinese-financed hydroelectric power plant development project in Nigeria’s Mambilla Plateau, involving multiple large dams, underground powerhouses, and an extensive high-voltage transmission network (ID: 7536)	Not relevant	Not relevant	Relevant	Relevant
14: Life Below Water	A $2 billion, 10-year sale-and-leaseback of Petrobras’ P-52 and P-57 offshore deep-water oil platforms in Brazil, with ownership reverting after the lease. (ID: 9600)	Relevant	Not relevant	Relevant	Not relevant
15: Life on Land	An $8 billion framework credit from China Development Bank to Vnesheconombank for infrastructure, agro-industrial, and communication technology projects in Russia’s Far East and Siberia, including housing, forestry, and electrolytic manganese initiatives.	Relevant	Not relevant	Relevant	Not relevant
16: Peace, Justice, and Strong Institutions	A record-breaking, long-term China–Russia oil supply and prepayment agreement, under which China National Petroleum Corporation advanced tens of billions of dollars to Rosneft in exchange for doubling crude deliveries through the Eastern Route pipeline over 25 years (ID:12443)	Relevant	Relevant	Not relevant	Not relevant
17: Partnerships for the Goals	A $50 billion syndicated loan from China Development Bank and Industrial and Commercial Bank of China to Indonesian state-owned enterprises for infrastructure development (ID: 9906)	Relevant	Relevant	N/A	Not relevant

### Uncertainties and limitations

While this dataset provides a systematic and transparent approach to identifying climate-related development finance, several limitations remain. First, project-level documentation, although often the most accessible source, is sometimes incomplete or lacks detail, with descriptions that do not always clarify whether climate objectives are primary or secondary. Second, the dataset reflects financial commitments rather than actual disbursements or realized outcomes, and therefore does not directly capture the delivery or effectiveness of climate finance. Linking commitments with verified disbursement records or post-implementation evaluations could enhance the assessment of financial impact. Third, classification is conducted at the project level, which is the finest granularity available from the sources. Some projects include multiple sub-components targeting different objectives, and the dataset cannot disaggregate funding to the sub-project level. In addition, achieving accurate identification of climate-relevant projects, particularly those with moderate or multiple objectives, is strengthened by expert review and the use of a carefully curated benchmark database, which can also guide and validate machine-assisted classification approaches.

## Supplementary information

Supplementary Tables

## Data Availability

The datasets are available through Figshare in format of Excel file: 10.6084/m9.figshare.30015124.

## Code availability

In this study, we use the Python language to run the language models and draw all figures. All codes has been uploaded to Figshare^28^.
