# Supplementary material for: A Benchmark Dataset of Chinese Development Finance with Climate Relevance and SDG Annotations from 2000–2021
Source: Sci Data. 2026 Jan 20;13:277. doi: 10.1038/s41597-026-06605-9 (PMC12921263; doi:10.1038/s41597-026-06605-9)
Supplement: Supplementary file 1 — Supplementary Tables [file 41597_2026_6605_MOESM1_ESM.pdf]

Supplementary Table 1. Labelling methodology for Rio Markers

Sector	Rationale for scoring	Mitigation	Adaptation	Examples
Action relating to debt	Excluded from marking except for debt which can be specifically targeted to environmental purposes.	/	/	Credit loan for hydroelectric power plant construction project (mitigation 2)
**Agriculture, forestry, fishing**	**Mitigation**: Projects that increase the efficiency or sustainability of agricultural production to reduce emissions.<br>● *Agriculture*: Farming methods decrease GHG emissions or increase carbon sequestration.<br>● *Forestry*: Systematically replanted after the trees that have grown are cut to provide carbon sequestration benefits.<br>● *Fishing*: Introduction of the energy-efficient fishing carrier.<br>**Adaptation**: Agricultural development measures that increase resilience to the impacts of climate change.<br>● *Agriculture*: Use of climate-resilient crops or diversifying production to be able to better cope with the impacts of climate change.<br>● *Forestry*: Improved forest management and	0, 1 or 2	1, 2 or 0	● Livestock feed donation to the drought-affected areas (adaptation 2)<br>● Training of water-saving and drought-resisting rice technology (adaptation 2)<br>● Pump irrigation project to improve farm-to-market roads and drainage infrastructure (adaptation 1 or 2)<br>● Forestry plantation to reserve sufficient forest resources and development of agro-economy (mitigation 2 adaptation 1)

	reforestation/afforestation that enhance adaptation capacities.<br>● *Fishing*: Promoting changes in fishing practices to adapt to changes in stocks and species.<br><br>**Overlap**: Forestry/afforestation measures (scoring both as principal should be exceptional)			
Banking and financial services	Green credits designed for renewable energy, low-carbon investments, energy efficiency or climate adaptation qualify for the climate markers.	0, 1 or 2	0, 1 or 2	Loans that incentivize better climate change risk analysis and increase its funding for green projects (mitigation 2 or 1 adaptation 2 or 1)
Business and other services	**Mitigation**: Activities that include the provision of advice to businesses in greening their practices or incentives that include climate change concepts in their strategies or guide their investment.<br><br>**Adaptation:** Diversify income opportunities for communities that are greatly affected by climate change and support the development, distribution or adoption of new technologies to better deal with climate change.	1, 0 or 2	1, 2 or 0	● Sustainable operations in agriculture, fishery, forestry and tourism (adaptation 1)<br>● Private investment in electric/hybrid vehicles production (mitigation 2)<br>● Geothermal power assets (mitigation 2)

Communications	**Mitigation**: Communications technology that lead to mitigation efforts<br>**Adaptation:** Strengthening resilience of communication equipment	0, 1 or 2	0, 1 or 2	● Installation and access to the satellite television system in villages (mitigation 2 adaptation 1)<br>● Remote sensing satellite serving for meteorology, agriculture and natural disaster prevention purposes (adaptation 1)
Developmental food aid/food security assistance	**Adaptation:** Building of climate resilience in food production	0	0, 1 or 2	● Food aid for disaster relief for flood victims (adaptation 2)<br>● Food aid for disaster relief for victims affected by drought (adaptation 1)
Disaster Prevention and preparedness	**Mitigation**: Provision of service/tools to be better prepared in case of the occurrence of a disaster that leads to significant GHG emission reductions.<br>**Adaptation**: Activities that aim at reducing the vulnerability of the population, the economy, and its infrastructure against the short-term and long-term negative consequences of climate damage.	0 or 1	1, 2 or 0	● River deepening and sluice gate upgrading to reduce flooding (adaptation 2)<br>● New meteorological station donations (adaptation 2)<br>● Disaster relief supplies for drought (adaptation 1)

Education	**Mitigation**: Mitigation-oriented education programs and sustainable school facilities.<br><br>**Adaptation**: Adaptation-oriented education programs.	0, 1 or 2	0, 1 or 2	● Rural solar power photovoltaic equipment donation to university (mitigation 2)<br>● Research on renewable energy (mitigation 2)
Emergency response	The short-term response to support the affected population is designed with a clear link to climate change in terms of emission reduction or improvement of adaptive capacity.<br><br>The improvement of capabilities to cope with natural disasters caused by climate change in the aftermath of a natural disaster.	0 or 1	0, 1 or 2	● Recovery plan for the tsunami-affected area and promote eco-tourism (adaptation 1)<br>● Construction of the climate-smart school after the hurricane (adaptation 2)
**Energy**	**Mitigation**: Energy generation, distribution and efficiency projects that promote energy efficiency and renewable energy.<br><br>● *Energy generation*: Power generation projects in wind, photovoltaic or solar, geothermal, biomass, biogas, and ocean tide power. Hydropower projects are also included, except for negative climate impact demonstrated. All nuclear power	2, 1 or 0	0, 1 or 2	● Solar photovoltaic power generation systems to rural areas (mitigation 2)<br>● Construction of wind energy, photovoltaic and concentrated solar power, geothermal, biomass and biogas (mitigation 2 if main objective)<br>● Equipment for hydropower

	projects are excluded. Gas-related and other less GHG-intensive fossil fuel projects are also included.<br>● *Energy conservation and demand-side efficiency*: Retrofitting or the substitution of old technologies and prevent a long-term lock-in GHG-intensive infrastructure.<br>● *Energy policy, education, and research:* :Renewable energy transmission and trainings and research on clean energy or energy efficiency are included.<br><br>**Adaptation**: Specific measures or designs to increase the security of supply in case of extreme weather events and based on vulnerability assessments.			plant transmission line (mitigation 2)<br>● LNG vessel acquisition project (mitigation 1)
General budget support	Excluded from marking in terms of general budget support. Sector budget support can be marked	0 or 1	0 or 1	Funded projects include natural disaster protection, environmental conservation and forestry (mitigation 1 adaptation 1)
**General environmental protection**	**Mitigation**: Practices in biosphere protection and flood prevention that could reduce or store emissions.	2, 1 or 0	2, 1 or 0	● Scientific investigation of climate change and water resources (mitigation 2

	● *Biosphere protection/Biodiversity*: Preservation of the ecosystem with the CO2 storage capacity.<br>● *Flood prevention/control*: Measures that reduce the consumption of energy and reduce GHG emissions.<br><br>**Adaptation**: Practices in biosphere protection and flood prevention that are employed for adaptation to the impacts of climate change.<br><br>● *Biosphere protection/Biodiversity*: Climate-resilient conservation of oceans and other marine coastal environments, wetlands, wilderness. ecosystems and protected areas.<br>● *Flood prevention/control*: Flood and coastal protection as well as drainage measures, remote sensing and satellite technologies used to enhance sustainable forestry management, urban/agricultural planning, and drought prevention.<br><br>**Overlap**:<br><br>● Institutional reforms and strengthening to include			adaptation 2)<br>● Strengthen institutions and implement laws related to environmental protection (mitigation 2 adaptation 2)<br>● Biogas equipment to promote bio-energy equipment and utilization, and to improve rural ecological environment (mitigation 2 adaptation 1)<br>● Environmental monitoring equipment (mitigation 2)<br>● Construction of sea wall and boulder groynes and stone pitching of village drains to protect the village from high tides (adaptation 2)<br>● Biodiversity conservation activities within national park (adaptation 1)

	climate aspects in policies and regulation.<br>● Afforestation or re-introduction of native tree species.<br>● Environmental training and research.			
Government and civil society	Development programs that integrate climate change considerations or promote climate change action.	0, 1 or 2	0, 1 or 2	● Seminar on green development (mitigation 1 adaptation 1)<br>● Meteorological equipment donation (adaptation 2)<br>● Electric vehicle donation for public duties (mitigation 1)<br>● Training and workshops for protection of women-owned farms (adaptation 1)
Health<br>(Population policies / programmes and reproductive health)	**Mitigation**: Activities in the health sector that reduce greenhouse gas emissions.<br>**Adaptation**: Health activities that aims or helps to address the consequences of climate change in the health of the population.	0 or 1	0, 1 or 2	● Upgrade hospitals and local health centers in areas affected by flooding (adaptation 1)
Industry, mining, construction	**Mitigation**: Changes in the demand patterns influence the resource chain and have impacts on GHG emissions, e.g. improvements in processes and cleaner	0, 1 or 2	0, 1 or 2	● Construction of a photovoltaic power station to supply electricity to factories, schools and homes

	production.<br><br>**Adaptation**: Activities designed to include considerations of climate change impacts, for instance, the design of climate-resilient equipment.			(mitigation 2)<br>● Natural gas pipeline and storage construction (mitigation 1)<br>● Flood control projects that involved the construction of flood management systems (adaptation 1)
Other commodity assistance	Development programs that integrate climate change considerations or promote climate change action.	0 or 1	0 or 1	Solar or energy-saving lamps handover (mitigation 1)
Other Multisector	**Mitigation**: The project (rural development / urban development / other) gets scored if there are measures put in place to reduce the emissions of GHG.<br><br>**Adaptation**: The project that includes measures to increase resilience of population or ecosystems in rural or urban areas to climate change.	1, 2 or 0	1, 2 or 0	● Rebuild after natural disaster and help with rural development (adaptation 2)<br>● Solar water heaters (mitigation 2)<br>● Waste removal and treatment equipment and dam construction equipment (adaptation 1)
Other social infrastructure and services	**Mitigation**: Activities if there are measures put in place to reduce the emissions of GHG.<br><br>**Adaptation**: Activities dedicated to climate proofing social infrastructure and services can be considered.	0, 1 or 2	0, 1 or 2	● Solar-powered traffic lights (mitigation 2)<br>● Social housing unit and infrastructure that includes roads, water supply and drainage system, electric

				light systems, etc. (mitigation 1 adaptation 1)
Reconstruction relief and rehabilitation	**Mitigation**: Restoration of services or repairing of infrastructure with the incorporation of low carbon technologies.<br><br>**Adaptation**: Improvement of capabilities to cope with the natural disaster caused by climate change.	0 or 1	0, 1 or 2	● Build bridges to provide access to the flooded villages (adaptation 1)
Trade policies and regulations	Activities that focused on mitigating the causes of climate change or adapting to the effects of it.	0 or 1	0, 1 or 2	No examples
**Transport and storage**	**Mitigation**:<br><br>● *Transport and storage*: Activities targeted to supporting the development of transport sector policy and planning can incorporate measures to promote GHG emission reductions.<br>● *Road / Rail / Water/ Air Transport*: Activities in the transport sector that aims at reducing GHG emissions.<br><br>**Adaptation:**<br><br>● *Transport and storage*: Climate-proofing	1, 2 or 0	0, 1 or 2	● New energy buses (mitigation 2)<br>● Water damaged road repair (adaptation 2)<br>● LNG carrier new building (mitigation 1)

	transport infrastructure and strategies.<br>● *Road / Rail / Water/ Air Transport*: The measures that significantly improves the resilience of transportation routes to extreme weather events or gradual changes in climate.			
Water supply and sanitation	**Mitigation**:<br>● *Water supply and sanitation*: Activities in the provision of water and/or sanitation aims or helps to achieve significant energy savings.<br>● *Waste management/disposal*: Activities that promote modern waste-to-energy with waste collection/recycling and recovery/use of methane gas that can result in significant GHG reductions.<br>● *Water resources conservation*: Water resources conservation involving the efficient use of energy or including forest preservation or other activities that provide terrestrial carbon uptake benefits contribute to mitigation.<br>**Adaptation:**<br>● *Water supply and sanitation*: Activities that aim	0, 1 or 2	1, 2 or 0	● Water resource search project related to climate change mitigation and hydropower development (mitigation 1 adaptation 2)<br>● Microgrid seawater desalination project (mitigation 2 adaptation 1)<br>● Multi-purpose dam project for electricity generation and water supply (mitigation 1 adaptation 1)<br>● Climate-resilient water supply project (adaptation 2)<br>● Solar water pumping system (mitigation 1)

	or help to address the expected changes or fluctuations in water supply and sanitation services as a consequence of climate change.<br>● *Waste management/disposal*: Effective waste management systems that protect water resources or fragile ecosystems and strengthen their resilience to the impacts of climate change.<br>● *Water resources conservation*: Climate resilience activities if an assessment of climate change risks include water shortages or high fluctuations in available water resources.<br><br>**Overlap:** Installation of systems that enable significant energy savings compared to older systems may qualify as resource-efficient systems reduce emissions while building resilience.			

Supplementary Table 2. Labelling Methodology for SDG Markers

Goal	Sector (Concentrated)	Key Words (Aiddata-referenced CRS purpose codes /Toetzke et al., 2022)
Goal 1: No Poverty	General Budget Support; Health; Other Social Infrastructure and Services	social/ welfare services; disaster prevention and preparedness
Goal 2: Zero Hunger	Agriculture, Forestry, Fishing; Developmental Food Aid/ Food Security Assistance	basic nutrition; agriculture, agricultural policy and administrative management; agricultural development; agricultural land resources; agricultural water resources; agricultural inputs; agricultural education/training; agricultural research; agricultural services, purpose; forestry development; fishery development; fishing development; fishery education/training; fishery research; fishery services; food aid/food security programmes
Goal 3: Good Health and Well-Being	Health	health policy and administrative management; medical education/training; medical services; basic health care; basic health infrastructure; infectious & parasitic disease control; health education; health personnel development; population policies/ programmes and reproductive health, population policy and administrative management; reproductive health care; family planning; std control including hiv/aids; personnel development for population and reproductive health; industrial development
Goal 4: Quality Education	Education	education facilities and training; teacher training; primary education; basic life skills for youth and adults; early childhood education; secondary education; vocational training; higher education; advanced technical and managerial training; medical education/training; tourism policy and administrative management
Goal 5: Gender Equality		family planning; strengthening civil society; women's rights organisations and movements, and government institutions; ending violence against women and girls; social services (incl youth development and women+ children); education in emergencies
Goal 6: Clean Water and Sanitation	Water Supply and Sanitation; Transport and Storage; Energy	water supply and sanitation, water resources policy and administrative management; water resources protection; water supply and sanitation - large systems; basic drinking water supply and basic sanitation; river development
Goal 7: Affordable	Energy; Industry, Mining,	energy generation and supply, energy policy and administrative management; power generation/non-renewable

and Clean Energy	Construction	sources; power generation/renewable sources; electrical transmission/ distribution; gas distribution; petroleum distribution and storage; industrial development
Goal 8: Decent Work and Economic Growth	Industry, Mining, Construction; Banking and Financial Services;	economic and development policy/planning; child soldiers (prevention and demobilisation); employment policy and administrative management; banking and financial services; financial policy and administrative management; monetary institutions; formal sector financial intermediaries; informal/semi-formal financial intermediaries; education/training in banking and financial services; business support services and institutions; business education and training; agricultural services, purpose; forestry services; fishery services; small and medium-sized enterprises (sme) development; cottage industries and handicraft; tourism policy and administrative management; women in development; non-agricultural alternative development
Goal 9: Industry, Innovation and Infrastructure	Industry, Mining, Construction; Energy; Transport and Storage; Communications	education facilities and training; primary education; secondary education; higher education; basic health infrastructure; water supply and sanitation - large systems; river development; water research; government administration; social/ welfare services; road transport; rail transport; water transport; air transport; communications policy and administrative management; telecommunications; radio/television/print media; information and communication technology (ict); communications, education and training.; energy research; financial policy and administrative management; formal sector financial intermediaries; business support services and institutions; agricultural development; agricultural water resources; fishery services; industrial policy and administrative management; industrial development; small and medium-sized enterprises (sme) development; cottage industries and handicraft; industry education and training; technological research and development; industry services; mineral resources and mining; mineral/mining policy and administrative management; mineral/metal prospection and exploration; mining education / training; construction policy and administrative management; tourism policy and administrative management; urban development and management; rural development; import support (capital goods)
Goal 10: Reduced Inequalities		social/ welfare services; financial policy and administrative management
Goal 11: Sustainable Cities	Energy; Other Social Infrastructure and	waste management/disposal; social/ welfare services; housing policy and administrative management; transport policy and administrative management; road transport; rail transport; water transport; air transport; education and

and Communities	Services; Transport and Storage	training in transport and storage; agricultural development; biosphere protection; site preservation; flood prevention/control; urban development and management; rural development; disaster prevention and preparedness
Goal 12: Responsible Consumption and Production	Agriculture, Forestry, Fishing	agricultural services
Goal 13: Climate Action	Energy; General Environmental Protection; Industry, Mining, Construction; Transport and Storage	fossil fuel electric power plants with carbon capture and storage (CCS); hybrid energy electric power plants, solar energy for centralised grids, solar energy for isolated grids and standalone systems, Solar energy - thermal applications, wind energy, marine energy, geothermal energy, biofuel-fired power plants, hydro-electric power plants, natural gas-fired electric power plants; electrical transmission/ distribution; environmental education/training, environmental research; environmental policy and administrative management; biosphere protection; flood prevention/control
Goal 14: Life Below Water	Agriculture, Forestry, Fishing	fishing policy and administrative management; fishery development; fishery research; biosphere protection
Goal 15: Life on Land	Agriculture, Forestry, Fishing; General Environmental Protection	agricultural land resources; forestry policy and administrative management; forestry development; forestry education/training; forestry research; forestry services; bio-diversity
Goal 16: Peace, Justice, and Strong Institutions	Government and Civil Society	education policy and administrative management; primary education; secondary education; higher education; health policy and administrative management; population policy and administrative management; water resources policy and administrative management; economic and development policy/planning; public sector financial management; legal and judicial development; government administration; strengthening civil society; conflict prevention and resolution, security system management and reform; civilian peace-building, conflict prevention and resolution; post-conflict peace-building (un); reintegration and salw control; land mine clearance; child soldiers (prevention and demobilisation); social/ welfare services; employment policy and administrative management; housing policy and administrative management; transport policy and administrative management;

		communications policy and administrative management; information and communication technology (ict); energy policy and administrative management; electrical transmission/ distribution; financial policy and administrative management; monetary institutions; business support services and institutions; agricultural policy and administrative management; forestry policy and administrative management; fishing policy and administrative management; industrial policy and administrative management; mineral/mining policy and administrative management; construction policy and administrative management; trade policy and administrative management; trade facilitation; tourism policy and administrative management; environmental policy and administrative management; women in development; urban development and management; rural development; disaster prevention and preparedness
Goal 17: Partnerships for the Goals		population policy and administrative management; public sector financial management; social/ welfare services; business support services and institutions; trade policy and administrative management; trade facilitation; regional trade agreements (rtas); multilateral trade negotiations; rural development; export support; action relating to debt; debt forgiveness; relief of multilateral debt; rescheduling and refinancing
